# Who Is Most Likely to Experience Corruption When Seeking Healthcare in Nigerian Healthcare Facilities?

**DOI:** 10.34172/ijhpm.8687

**Published:** 2025-04-21

**Authors:** Ifunanya Clara Agu, Chukwudi Nwokolo, Obinna Onwujekwe, Martin McKee, Eleanor Hutchinson, Blake Angell, Dina Balabanova

**Affiliations:** ^1^Health Policy Research Group, College of Medicine, University of Nigeria, Enugu, Nigeria.; ^2^University of Nigeria, Nsukka, Nigeria.; ^3^London School of Hygiene and Tropical Medicine, London, UK.; ^4^University of New South Wales Sydney, The George Institute for Global Health, Newtown, NSW, Australia.

**Keywords:** Health Services, Patients Corruption Experience, Public Healthcare Facilities, Nigeria, Corruption

## Abstract

**Background:**

Experiencing corruption when seeking health services remains a significant problem in Nigeria. An effective response requires knowledge of the individual characteristics of those impacted by corruption when seeking healthcare. This study examined the prevalence of corruption among those seeking health services in Nigeria’s public healthcare facilities and how it varies among different user groups.

**Methods:**

We used a pre-tested interviewer-administered questionnaire to collect data from 1659 individuals randomly selected from households in two Nigerian states. We collected data on respondents’ socio-demographic characteristics and experiences of corrupt practices. We undertook descriptive and binomial logistic regression analyses.

**Results:**

Approximately 50% (823) of respondents experienced corrupt practices, such as using connections for faster treatment and bribery when seeking health services. 446 (27%) respondents bribed or made so-called unapproved payments to health providers to obtain health services. Gender was a strong predictor, with male healthcare service users being more likely to experience corrupt practices (%point risk difference=24; 95% CI=20, 29) and bribe or make an unapproved payment to obtain healthcare (%point risk difference=20; 95% CI=15, 25). Residents in the northern state were (%point risk difference=30; 95% CI=26, 35) more likely to experience corrupt practices than residents in the eastern state. People seeking healthcare in urban (%point risk difference=09; 95% CI=-05, 08) and semi-urban (%point risk difference=12; 95% CI=05, 19) locations were more likely to have bribed or made ‘unapproved’ payments to healthcare providers compared to rural residents.

**Conclusion:**

Health sector corruption, in its various forms, is frequently reported in both northern and southern Nigeria. However, user experience of corruption varies according to socio-demographic characteristics, and this is often insufficiently acknowledged. To combat corrupt practices in both health sectors, anti-corruption initiatives must be tailored to particular groups and settings, addressing specific disadvantages at individual and community levels.

## Background

**Key Messages**

**Implications for policy makers**

- Corruption affects health service users differently across states, suggesting the need for region- or context-specific interventions. Policy-makers should design targeted strategies that allocate resources to strengthen governance and address the unique challenges service users face in different geographical areas.
- Male healthcare service users are more likely to engage in corrupt practices, as are those with higher levels of education. This insight can help policy-makers create more targeted, gender-sensitive policies to curb corruption. Awareness campaigns and behavioural interventions should focus on educating male and higher educated users about the negative impacts of corruption and fostering a culture of accountability.
- Individuals receiving care from doctors or medical officers were more likely to experience corruption, highlighting the need for policy interventions that can account for more powerful groups within the system.
- Our findings on generational and demographic disparities in corruption experiences underline the need for policies catering to different populations’ specific needs, and understanding how corruption affects various age groups and demographics is crucial for developing inclusive policies.
- Corruption is a significant issue in the public and private healthcare sectors, requiring stronger regulatory frameworks. Policies should involve the private healthcare sector in anti-corruption initiatives to improve service quality and reduce corruption across all healthcare facilities.

**Implications for the public**

Our study provides critical insights into the pervasive corruption issue within Nigeria’s healthcare system, highlighting varied experiences of healthcare service users in Eastern and Northern states. These findings demonstrate that corruption is not uniform; it manifests differently depending on regional and socio-economic contexts. Addressing these varied experiences requires tailored community engagement initiatives that empower individuals to demand transparency and accountability from healthcare providers. Involving community leaders and influencers can drive collective action against corruption and encourage reporting of observed corrupt practices at healthcare facilities. Additionally, public awareness campaigns and educational programs are crucial for informing the public about their rights and proper channels for addressing grievances related to healthcare services. These efforts can help to improve access to quality care, enhance health outcomes, and rebuild public trust in the healthcare system, ultimately benefiting all health service users.

Corruption in health systems has numerous harmful consequences, disproportionately affecting the most vulnerable populations by restricting their access to essential healthcare services.^1-3^ Transparency International defines corruption as “the abuse of entrusted power for private gain,” which includes practices such as embezzlement, favouritism, and bribery.^4^ These practices undermine both access to healthcare and the quality of services provided.^5^

 In sub-Saharan Africa, individuals who paid bribes for healthcare were four to nine times more likely to experience difficulties accessing services.^6^ Corruption also discourages investment in health systems.^2,7^ The consequences are severe: lack of timely care can result in death,^8,9^ with an estimated 140 000 annual deaths among children under five attributed to corruption in health systems.^10^ Other negative outcomes include reduced immunisation rates,^11^ increased antibiotic resistance,^12^ and poorer mental health among service users.^13,14^ A recent study analysing data from 17 sub-Saharan African countries found a strong link between higher levels of corruption and bribery in health services and increased maternal mortality rates.^15^

 Corruption manifests in various forms and occurs at all levels within health systems, from government officials to frontline healthcare providers.^16^ Motivations for engaging in corrupt practices vary, but their effects are consistently damaging.^17^ People impacted by corrupt practices—such as absent doctors, demands for informal payments, drug shortages, long wait times, and lack of transparency regarding service costs—are more likely to report paying bribes.^18,19^ Informal payments, referred to as “unapproved payments” in this study, are unofficial fees charged by healthcare providers to deliver entitled services or offer preferential treatment.^20,21^

 Corruption significantly impacts the healthcare sector in many African countries. Evidence from a study conducted in Ghana, Kenya, and Uganda highlights that illegal payments are a common requirement for accessing healthcare services.^22^ This situation mirrors the challenges faced in Nigeria, where corruption in the healthcare system is widespread.^21,23-25^ A study in sub-Saharan Africa found a high incidence of patients having to pay bribes for medical care in several countries.^6^ For instance, in Liberia, 53% of respondents reported paying bribes, while the figures were 32% in Sudan, 30% in Cameroon, 25% in Guinea, and 25% in Sierra Leone.^6^

 Nigeria’s healthcare system operates through both public and private sectors, which aim to meet the diverse health needs of the populace. The public sector provides subsidised services but is plagued by inefficiencies, inadequate infrastructure, and workforce challenges.^26,27^ In contrast, the private sector is better resourced but is less accessible to lower-income groups due to its higher out-of-pocket costs.^26,27^ Recent documentation reported that less than 5% of Nigerians have access to formal health insurance coverage.^28,29^ The National Health Insurance Authority aims to achieve universal health coverage, but out-of-pocket payments still account for about 70% of healthcare spending.^29,30^

 Corrupt practices in Nigeria’s healthcare system manifest in various forms, including absenteeism, unapproved payments, under-the-counter payments, health financing-related corruption, and abuse of power in employment and pharmaceutical procurement.^24,31,32^ Factors such as inadequate salaries, shortage of medical supplies, substandard working environment, and poor monitoring structure were identified as key drivers of corrupt practices in both Nigeria and Ghana.^21,23,33^

 In Nigeria, health workers face sub-optimal economic conditions due to insufficient financial resources to meet their basic needs and earnings that do not commensurate with their work efforts, compelling them to consider other income-earning options.^23^ This has been a major contributor to the prevalent corrupt practices such as absenteeism and unapproved payments, recorded in public healthcare.^23^ These corrupt practices undermine the quality of healthcare services, increase out-of-pocket costs among users, lead to poor health outcomes, and erode public trust in the healthcare system.^20,21^

 Nigerian authorities have established the Economic and Financial Crimes Commission and the Independent Corrupt Practices and Other Related Offences Commission to address corruption. These bodies are empowered to investigate and prosecute financial crimes and corrupt practices, including those in the health sector.^34^

 Despite these efforts, there is limited understanding of who is most affected by corruption when accessing public healthcare facilities in Nigeria. Research has explored the extent and nature of corrupt practices, their impact on healthcare delivery, and the development of anti-corruption strategies.^24,32,35^ However, these studies emphasise the need to address underlying factors that may enable or constrain the success of these measures.

 This study contributes to addressing this gap by presenting findings from a survey of healthcare users across different system levels. The survey examines the relationship between users’ characteristics and their experiences when seeking care, providing new insights into the groups most affected by corruption in public health facilities.

## Methods

###  Study Design, Study Area, and Household Selection 

 Our study was part of a broader research project on drivers of corruption, Accountability in Action.^36^ Qualitative methods, including ethnographic participant observation and in-depth interviews conducted before the survey, informed the questionnaire design.^21^ This quantitative cross-sectional study was carried out in two Nigerian states in 2022. Nigeria has three major ethnic groups: the Igbo and Yoruba in the South and the Hausa in the North. One of the selected states is in the eastern region, predominantly Igbo, while the other is in the northern region, predominantly Hausa.

 These states were purposefully chosen based on our deep contextual knowledge, which supported survey design, and our long-standing collaborations with research institutions and policy-makers addressing illicit practices in health systems. The states also reflect diverse sociocultural factors, such as education and religion, that influence corrupt behaviors, making them ideal for the study.

 We employed multi-stage sampling to select households.^37^ In the first stage, we selected the two states as described. In the second stage, we purposively selected at least three local government areas within each state to ensure diverse representation across urban, semi-urban, and rural areas. Eligible communities—those with public health facilities—and households were prioritised. Geopolitical diversity within each state was also considered, while locations with significant security risks were excluded from the sampling process.

 In the third stage, we employed a modified consecutive sampling method to select eligible households, starting from the public health facility in the community. Households were considered eligible if they had at least one member aged 18 to 65 who had lived in the community for at least six months and had sought care from a public health facility within the six months preceding the interview. If a household did not meet these criteria, it was replaced until the required sample size was achieved. Within each household, the primary caregiver was the preferred respondent. If the primary caregiver was unavailable, another family member with adequate knowledge of the household’s healthcare utilisation was interviewed. We assigned unique codes to each household and respondent to maintain anonymity and confidentiality, ensuring that personal identifiers were not linked to the data.

###  Study Population

 In this study, *health service users* are defined as individuals (or members of their households) aged 18 to 65 who accessed services at a public healthcare facility—whether primary, secondary, or tertiary—within the six months preceding the survey. These visits could include various health needs, such as treatment, preventive services like vaccinations or prenatal care, initial consultations, diagnostic tests, routine check-ups, obtaining medical certificates, or other health-related purposes. Individuals with neurocognitive impairments that prevented participation were excluded from the study. Those who met the eligibility criteria and consented to participate were also asked if they had used a private healthcare facility in addition to the public facility within the past six months.

###  Data Collection

 Data collection took place in November and December 2022, yielding a final sample of 1659 households. The initial draft of the structured questionnaire used in this study was adapted from a previously published study.^21,24^ This draft was reviewed and refined with key stakeholders to ensure its relevance and accuracy.

 Interviews were conducted by a team of 80 trained research assistants working in pairs, overseen by 12 supervisors. The research assistants, all with at least secondary education, were familiar with the locations where they collected data, including knowledge of the local dialects. Depending on the location, interviews were conducted in English, Hausa, Igbo, or Pidgin. Responses were recorded in duplicate: electronically using ODK software on tablets and manually on paper questionnaires. Any discrepancies were reconciled with the field supervisor at the end of each day. The electronic data were uploaded to a server and then downloaded into Excel and SPSS after fieldwork for viewing and cleaning. The cleaned data were imported into Stata for analysis.

###  Description of Variables 

 This study used two outcome variables to measure corruption: (*i*) participants’ experience of rule-breaking corrupt practices and (*ii*) “unapproved payments” to obtain services.

 Participants’ experiences of rule-breaking, corrupt practices were captured from responses to the question: *“Did you or your family member experience any of the following when receiving services or attending a public facility for a different reason?”* Possible responses were: one or more persons jumping the queue (eg, obtaining priority service because they know staff); you were asked to bring excessive amounts of consumables; you had to pay higher fees than the advertised price for services; fewer than required or no health workers were available to provide services; the facility was closed when it should be open; you had to give favours or services to the facility or health worker to get medical attention; health workers were free but refused to attend to you because you refused to tip them.

 In this study, the second outcome variable, *unapproved payment*, refers to participants’ unofficial payment or favours to health facility staff to access or utilise healthcare services. During the survey, these payments were described as bribes, favours, or gifts provided to staff in exchange for health services. To capture this variable, participants were asked: *“During your most recent visit to a public health facility within the last six months, if you made an unapproved payment, gave a gift, bribe, or did a favour for a staff member, when or at what stage of the service delivery did this occur?”*

 Participants could choose one of the following options: Before the service was delivered; After the service was delivered; At the same time the service was delivered; Partly before and partly after the service was delivered; Nothing was paid; Not sure/do not know.

 The independent variables used to assess the outcome included age, gender, state, geographical location of residence (urban/rural), perceived sufficiency of household income relative to needs, highest educational qualification, the cadre of the health provider delivering the service, and the type of health facility utilised. These variables were selected because they reflect an individual’s biological differences, environmental exposures, health literacy, expertise of healthcare providers, and economic stability of a household, all of which were found to influence behaviors and access to quality healthcare.^38-41^

 Respondents’ ages at the time of the survey were categorised into generational cohorts: *Baby Boomers* (1928–1964); *Generation X* (1965–1980); *Generation Y/Millennials* (1981–1997); *Generation Z* (1998–2012).^42-45^ Age was included as one of the independent variables due to different age groups having varying health needs and risks, making it crucial to understand how age affects health outcomes.^41^

###  Data Analysis 

 We analysed the data using the STATA statistical package version 17. The first step involved univariate analysis, reporting frequencies and percentages for categorical variables and means and standard deviations for continuous variables.

 To assess each specific dependent variable (“experience of rule-breaking corrupt practices” and “unapproved payment”), individual responses were scored as “1” if the participant reported any corrupt practices experienced and “0” for “no” (none of these/nothing was paid, or not sure/do not know).

 We used bivariate logistic regression to calculate 95% confidence intervals (CIs) on the log-odds scale using the Wald statistic, which was then converted to a probability scale. The risk differences were presented as percentage points using binomial regression models. These are generalised linear models with identity links and binomial errors. Each response was treated as a binary outcome, with a single independent (dummy) variable code used for the relevant comparison.

## Results

Table 1 describes the characteristics of the participants. One thousand six hundred fifty-nine individuals participated in the survey, comprising 659 men and 1000 women who used public healthcare services in the two Nigerian states within the six months preceding the survey. Fifty-two percent (864) reported that they find it difficult to sustain their current household incomes, while 30% reported living comfortably. Slightly over half (844) of respondents resided in rural locations, 10% were semi-urban residents (176), and 38% lived in urban locations. Many respondents sought healthcare from 857 (51%) primary health centres and district health facilities 552 (33%).

**Table 1:** Socio-demographic Characteristics of Participants in the Survey

**Variable (N = 1659)**	**Frequency **	**Percent **
Gender		
Male	659	39.72
Female	1000	60.28
State of residence		
Eastern state	831	50.09
Northern state	828	49.91
Location of residence		
Urban	639	38.52
Semi-urban	176	10.61
Rural	844	50.87
Age category		
Boomers (≥58)	162	9.76
Gen X (42–57)	339	20.43
Gen Y (25–41)	969	58.41
Gen Z (18–24)	189	11.39
Mean age (SD) = 37.39 (12.71); Median age (IQR) = 35 (28-44)		
Highest educational level/qualification		
No formal education	144	8.68
Primary	148	8.92
Secondary	791	47.68
University	244	14.71
Diploma (HND/NCE)	299	18.02
Others (Islamic school)	33	1.99
Perceived sufficiency of household income		
Living comfortably on current income	172	10.37
Getting by on current income	864	52.08
Finding it very difficult on current income	623	37.55

Abbreviations: SD, Standard deviation; IQR, interquartile range; HND, Higher National Diploma; NCE, Nigeria Certificate in Education.

Table 2 shows the type of health facility and providers that treated the respondents. The most common case is for the respondents to receive care from a nurse during their last visit to a public facility (46%). Many respondents sought healthcare from 857 (51%) primary health centres and district health facilities 552 (33%).

**Table 2:** Type of Health Facility and Providers Who Provided Healthcare Services for the Survey Participants

**Variable (N = 1659)**	**Frequency**	**Percent**
**Public healthcare facility **		
Tertiary	206	12.42
Secondary/district	552	33.27
Primary health centre	857	51.66
Health post	42	2.53
Not sure/others	02	0.12
**Private healthcare facility **		
Private-for-profit clinic or hospital	420	25.32
Non-governmental (eg, faith-based or mission hospital)	122	7.41
Individual private practice-midwife/nurse/doctor	158	9.52
Traditional healer	56	3.38
Only sought care in a public facility	809	48.76
Not sure/do not know	56	3.38
Other	37	2.23
**Health service provider **		
Doctor	535	32.44
Nurse	769	46.63
Midwife	87	5.28
Community health extension worker	21	1.27
Medical officer/ad hoc	231	14.01
Not sure/do not know	06	0.36

Abbreviation: CHEW, Community health extension worker.

 Across the two states, approximately 50% of respondents reported experiencing corrupt practices when seeking healthcare (See Table 3). Among those, 13% (217) reported queue jumping and seeing other health service users given priority because they knew the health workers, 12% (199) provided favours or services to staff to get medical attention, and 9% (146) reported that there were few or no available health workers in the facility. Twenty-seven percent (446) of respondents made informal payments or bribed the provider(s) to access healthcare.

**Table 3:** Description of Participants’ Experiences and Observations of Rule-Breaking/Corruption While Seeking Health Services in Public Health Facilities

**Variable (N = 1659)**	**Frequency **	**Percent**
Participants or family members who experienced rule-breaking corruption		
Jumping the queue/obtaining priority service because they know the staff	217	13.08
Were asked to bring excessive amounts of consumables	86	5.18
Paid higher fees than the actual price for services	96	5.79
Fewer than required or no health workers were available to provide services	146	8.80
Facility was closed when it should have been opened	57	3.44
Provide favours or services to health workers to get medical attention	22	1.33
Health workers were free but refused to attend to the user because the user refused to tip them	199	12.00
None of these	797	48.04
Not sure/do not know	39	2.35
Number of participants who experienced any form of corruption		
Experienced corruption	823	49.61
Did not experience corruption	836	50.39
Bribery/“unapproved payments” to obtain services		
Before the service was delivered	218	13.14
After the service was delivered	94	5.67
At the same time that the service was delivered	82	4.94
Partly before and partly after the service was delivered	52	3.13
Nothing was paid, but payment is expected to happen in the future	235	14.17
Not sure/do not know	978	58.95
Number of participants who bribed/made unapproved payments		
Bribed/made “unapproved payment”	446	26.88
Did not bribe/made “unapproved payment”	1213	73.12

Table 4 presents a bivariate logistic regression of demographic factors associated with healthcare health service users’ experience of corruption and “unapproved payments.” Male healthcare service users were more likely to experience any form of corrupt practices (Percentage point risk difference 24; 95% CI: 20, 29) and bribed/ made “unapproved payments” (Percentage point risk difference 20; 95% CI: 15, 25). Healthcare service users who live in the northern state were (Percentage point risk difference 30; 95% CI: 26, 35) more likely to report experiencing corrupt practices and 6% likely to make “unapproved payments” (Percentage point risk difference 06%; 95% CI: 02, 10). The older generational cohorts were more likely to experience corruption and make “unapproved payments” than the youngest. Specifically, Generation Y, Generation X, and Boomers were more likely to make “unapproved payments” than Gen Z, with risk percentage differences of 5%, 7%, and 12%, respectively.

**Table 4:** Analysis of the Demographic Differences in Participants’ Experience of Corruption

	**Experienced Rule-Breaking, Corrupt Practices**		**Bribery/**“**Unapproved Payments” to Obtain Services**	
	**% (95% CI)**	**%Risk Difference (CI)**	**% (95% CI)**	**%Risk Difference (CI)**
Gender				
Male	64% (61%, 68%)		39% (35%, 43%)	
Female	40% (37%, 43%)		19% (16%, 21%)	
Male vs female		24% (20%, 29%)		20% (15%, 25%)
State of residence				
Eastern state	34% (31%, 38%)		24% (21%, 27%)	
Northern state	64% (62%, 68%)		30% (27%, 33%)	
Northern vs Eastern state		30% (26%, 35%)		06% (02%, 10%)
Location of residence				
Rural	47% (43%, 50%)		22% (19%, 25%)	
Semi-urban	38% (30%, 45%)		23% (17%, 29%)	
Urban	57% (53%, 60%)		35% (31%, 38%)	
Urban vs rural		10% (05%, 15%)		09% (-05%, 08%)
Urban vs semi-urban		19% (11%, 27%)		13% (08%, 17%)
Semi-urban vs rural		-09% (-17%, -01%)		12% (05%, 19%)
Age category				
Gen Z (18–24)	37% (30%, 44%)		21% (15%, 27%)	
Gen Y (25–41)	47% (44%, 50%)		26% (24%, 29%)	
Gen X (42–57)	63% (58%, 68%)		28% (24%, 33%)	
Boomers (≥58)	54% (46%, 61%)		33% (26%, 41%)	
Gen Y vs Gen Z		10% (02%, 17%)		05% (-01%, 12%)
Gen X vs Gen Z		26% (18%, 35%)		07% (-00%, 15%)
Boomers vs Gen Z		17% (06%, 27%)		12% (03%, 21%)
Gen X vs Gen Y				02% (-04%, 07%)
Boomers vs Gen X				05% (-04%, 14%)
Highest educational qualification				
None (N)	08% (08%, 24%)		17% (08%, 27%)	
Primary (P)	10% (14%, 25%)		45% (34%, 56%)	
Secondary (S)	45% (21%, 49%)		43% (37%, 48%)	
BD	15% (23%, 43%)		37% (28%, 45%)	
Diploma (D)	21% (12%, 29%)		30% (23%, 37%)	
P vs N		05% (-13%, 24%)		28% (13%, 42%)
S vs N		20% (-15%, 28%)		25% (14%, 36%)
BD vs N		18% (04%, 19%)		19% (06%, 32%)
D vs N		15% (01%, 27%)		13% (01%, 24%)

Abbreviations: CI, confidence interval; BD, Bachelor’s degree. *Note*: The 95% CIs are determined using a “logit” method. This involves computing the CIs on the log-odds scale through a logistic regression model, based on the Wald statistic, and then converting them to the probability scale. Risk differences are presented in percentage points and are calculated using binomial regression models (generalised linear models with identity links and binomial errors). Each response is treated as a binary outcome.

Table 5 presents data on the level of healthcare service users’ experience of corruption and “unapproved payment” disaggregated by their household income sufficiency, healthcare facility type and healthcare service providers. Fifty-one percent of service users who reported finding it very difficult to manage their current household income experienced corrupt practices (95% CI: 42, 60). Thirty-nine percent of them (95% CI: 30, 48) bribed or made “unapproved payments” to health facility staff to access health services. Approximately 46% of health service users who utilised a public health facility and half (54%) who accessed healthcare in a private health facility experienced corruption. Participants whose healthcare services were provided by a doctor were 21% more likely to experience corruption (95% CI: -01, 42) and 19% more likely to bribe or make “unapproved payments” (95% CI: 03, 34).

**Table 5:** Healthcare Service Users’ Experience With Corrupt Practices and Unapproved Payments Disaggregated by Perceived Household Income Sufficiency, Health Facility Type, and Health Service Providers

	**Experienced Rule-Breaking, Corrupt Practices**		**Bribery/**“**Unapproved Payments” to Obtain Services**	
	**% (95% CI)**	**Percentage Point Risk Difference (95% CI)**	**% (95% CI)**	**Percentage Point Risk Difference (95% CI)**
Perceived sufficiency of household current income				
LC	51% (43%, 58%)		40% (33%, 47%)	
GB	49% (46%, 52%)		23% (20%, 26%)	
FD	50% (46%, 54%)		26% (22%, 30%)	
FVD	51% (42%, 60%)		39% (30%, 48%)	
LC vs GB		02% (-07%, 10%)		17% (09%, 25%)
LC vs FD		01% (-08%, 09%)		14% (06%, 22%)
LC vs FVD		-00% (-12%, 11%)		-03% (-10%, 13%)
FVD vs FD		01%(-09%, 11%)		13% (0.3%, 22%)
Healthcare facility				
Public	46% (42%, 49%)		18% (16%, 21%)	
Private	54% (51%, 58%)		37% (34%, 41%)	
Private vs public		09% (04%, 13%)		19% (15%, 23%)
Public healthcare facility				
THF	63% (57%, 70%)		28% (22%, 34%)	
DHF	62% (58%, 66%)		36% (32%, 40%)	
PHC	38% (35%, 41%)		21% (18%, 23%)	
HP	55% (35%, 41%)		31% (17%, 45%)	
THF vs DHF		01% (-07%, 09%)		-09% (-15%, -01%)
THF vs PHC		25% (18%, 33%)		08% (01%, 14%)
THF vs HP		08% (-08%, 25%)		-03% (-18%, 12%)
Health service provider				
Doctor	64% (60%, 68%)		33% (29%, 37%)	
Nurse	37% (34%, 41%)		22% (19%, 25%)	
Midwife	47% (37%, 58%)		29% (19%, 38%)	
CHEW	43% (22%, 64%)		14% (-01%, 29%)	
Medical officer/ad hoc	60% (54%, 66%)		27% (22%, 33%)	
Not sure/do not know	50% (10%, 90%)		50% (10%, 90%)	
Doctor vs CHEW		21% (-01%, 42%)		19% (03%, 34%)
Nurse vs CHEW		-06% (-27%, 16%)		08% (-08%, 23%)
Midwife vs CHEW		04% (-19%, 28%)		14% (-03, 32%)
Medical officer/ad hoc vs CHEW		17% (-05%, 39%)		13% (-03, 29%)
Nurse vs doctor		-26% (32%, 21%)		-11% (-16%, -06%)
Midwife vs doctor		-17% (-28%, -05%)		-05% (-15%, 06%)
Midwife vs nurse		10% (-01, 21%)		07% (-03%, 17%)

Abbreviations: CI, confidence interval; LC, living comfortably; GB, getting by; FD, finding it difficult; FVD, Finding it very difficult; THF, tertiary healthcare facility; DHF, district healthcare facility; PHC, primary health centre; HP, health post; CHEW, Community health extension worker. *Note*: The 95% CIs are determined using a “logit” method. This involves computing the CIs on the log-odds scale through a logistic regression model, based on the Wald statistic, and then converting them to the probability scale. Risk differences are expressed in percentage points and are calculated using binomial regression models (generalised linear models with identity links and binomial errors). Each response is treated as a binary outcome.

## Discussion

 We investigated the prevalence of corruption among individuals seeking healthcare services in Nigeria, examining how experiences vary across different groups. Corruption is increasingly recognised as a significant barrier to effective and equitable healthcare. Our findings show that healthcare service users experience and engage in corruption differently, with disparities shaped by factors such as geographic location, gender, generational cohort, and the type of healthcare facility accessed. These results highlight corruption’s complex and multifaceted nature in health systems and emphasise the need to understand how user experiences differ. Previous research underscores the importance of innovative anti-corruption strategies that consider systemic and contextual factors influencing healthcare corruption.^31,46,47^ For example, findings from a national survey indicate the potential success of systemic interventions, with consistently low corruption rates reported following the implementation of targeted anti-corruption campaigns.^47^

 This study revealed how most experienced corruption, taking multiple forms, when accessing care, including queue-jumping, a shortage or absence of health workers, and unapproved payments—either informally required or exceeding official fees. These findings affirm the urgent need to address diverse forms of corruption in the health sector by identifying their specific drivers and focusing on the experiences of the most affected groups.

 Residents of Nigeria’s northern region were more likely to encounter corrupt healthcare practices than those in the eastern region. This regional disparity reflects broader socioeconomic and governance challenges. Higher corruption levels in the northern region may be linked to factors such as weaker institutional frameworks and higher poverty levels.^48,49^ Additionally, the northern region has historically shown lower healthcare utilisation rates,^49^ a situation that the higher prevalence of corruption may further exacerbate. Corruption in healthcare settings compounds existing barriers to accessing and using formal health services, deepening health inequities in the region.^49^ Addressing these disparities requires region-specific anti-corruption strategies, including strengthening governance structures, allocating targeted resources, and implementing accountability mechanisms tailored to local contexts.

 Our study identified gender as a significant predictor of experiencing corrupt practices in public health facilities, with males being more likely than females to encounter such practices when seeking healthcare. This is in line with Afrobarometer analysis.^50^ However, evidence is somewhat mixed with other studies reporting that females are more likely to observe and experience corruption, albeit in different ways from males, reflecting broader unequal power dynamics between genders.^51,52^ Alatas et al found that females in Australia are less tolerant of corrupt practices compared to their male counterparts, while no significant gender differences were noted in India, Indonesia, and Singapore.^52^ This suggests that cultural factors and social norms play a crucial role in shaping attitudes towards corrupt practices, but this may be unique to each context. While corruption can hinder access to quality health services for all users, research indicates that women are disproportionately affected due to their more frequent interactions with the health system, for example, related to reproductive and child health needs.^21,53,54^ On the other hand, their increased familiarity with the healthcare system, resulting from their frequent use of services, could potentially mitigate the impact of corrupt practices on their access to care. Addressing gender-specific vulnerabilities and considering cultural contexts when developing effective anti-corruption strategies are important.

 We also found that males were more likely to report engaging in bribery or making “unapproved payments” to access healthcare services. This aligns with a study from Ghana, which noted that women are generally less likely to offer bribes, although that study did not focus specifically on healthcare-related bribery.^55^ The observed gender disparity may be explained by societal norms that position men as primary decision-makers, making them more frequent targets for corrupt demands.^56,57^ Healthcare providers may perceive men as more capable of meeting “unapproved payment” demands while viewing women as having fewer financial resources.^51,54^ This perception can place additional financial pressures on men, potentially obstructing them from seeking care for themselves and their families, thus worsening health outcomes. Conversely, some women who cannot afford bribes or excess fees, particularly for maternal and child health services, may resort to alternative care from informal or unlicensed providers, as reported in a recent Nigerian study.^21^ These findings highlight the importance of implementing gender-sensitive interventions that address the distinct vulnerabilities faced by men and women in healthcare settings while also tackling systemic inequities that enable corrupt practices.

 Our analysis revealed that older generational cohorts—Generation Y, Generation X, and Baby Boomers—are more likely to experience corrupt practices and engage in “unapproved payments” when seeking healthcare, compared to Generation Z. While these findings may reflect generational differences in exposure to anti-corruption campaigns, another possible explanation is that healthcare providers perceive younger individuals as having less income and access to financial resources, making them less likely targets for soliciting “unapproved payments.” An individual’s socioeconomic status and life stage can significantly influence their vulnerability to corruption.^58,59^ Younger generations are often seen as having fewer familial responsibilities, so they may be less likely to be in situations where “unapproved payments” are demanded. These findings underscore the need to address healthcare providers’ behaviors and perceptions in anti-corruption initiatives. Incorporating generational differences into policy design is essential. Training programs for healthcare providers should include components that challenge biases related to health service users’ demographics, ensuring ethical standards are upheld consistently across all age groups.

 We also found that urban residents are likelier to experience corruption and make bribes or “unapproved payments” to access health services. This aligns with research by Mangafić and Veselinović, which found that urban residents are more likely to engage in bribery, though their study focused on differences across sectors.^60^ A Nigerian study reported that health service users in wealthier urban health facilities are asked for higher unapproved payments than those attending rural facilities.^21^ This is a concerning issue that could negatively impact the health of the urban population, highlighting the need for actionable policies that promote transparency and accountability in urban public health systems.

 Our findings indicate that better-educated health service users are likelier to engage in bribery or make “unapproved payments.” Specifically, health service users with a diploma were fifteen times more likely to experience corrupt practices and thirteen times more likely to bribe or make “unapproved payments” at public healthcare facilities than those without formal education. This contrasts with the findings of Forson and colleagues, who found that education attainment has a significant positive effect on participation in corruption at the individual level.^61^ Walton and Perffer also reported that individuals with higher levels of education are more likely to recognise and report corrupt practices.^62^

 Our results suggest that while higher education increases awareness of corruption, it may also inadvertently increase the likelihood of engaging in such practices. This could be because better-educated individuals feel more capable of navigating the system and using corruption to their advantage. They may also better understand their rights and the workings of the healthcare system, or healthcare providers may perceive them as having higher incomes, making them more likely targets for corruption. Previous research has shown that educated people tend to use healthcare services more frequently and interact with public officials more often, providing more opportunities for bribery.^63,64^ Additionally, more educated individuals often place a higher value on their time, which could incentivise them to engage in corrupt practices to expedite care. To address this, revising the education curriculum to include lessons on the consequences of engaging in corrupt practices could help reduce such behaviors among the educated population. Strengthening patient advocacy programs and awareness campaigns targeting this group could discourage reliance on unapproved payments.

 Health service users who felt that their household income was sufficient for comfortable living during the survey were less likely to bribe or make “unapproved payments” than those who found it difficult to make ends meet. This may be because lower-income individuals may perceive bribery or unapproved payments as necessary to access essential services, potentially because they lack the power, confidence, or alternatives to navigate corrupt systems without paying. Economic stability may empower health service users to resist exploitative practices. Ogbozor et al found that there are generally no exemptions from “unapproved payments” when accessing maternal and child health services, regardless of income.^21^ This results in increased out-of-pocket expenses for both wealthy and low-income health service users, and those unable to make these payments are often denied healthcare.^21^ Public health facility managers and workers argue that “unapproved payments” are necessary to cover operational costs.^21^ Agwu and colleagues suggest that addressing healthcare corruption requires long-term interventions that focus on systemic healthcare flaws rather than only disciplining individual healthcare workers.^31^ Improved governance, better regulation, and adequate funding of healthcare services are essential for addressing the root causes of bribery and corruption.

 Corruption is also prevalent in private healthcare facilities, where 54% of health service users reported paying bribes and 37% made “unapproved payments.” This challenges the idea that formalising payments can reduce the incentives for bribery and unapproved payments. Health service users who face difficulties accessing services in public healthcare may pay bribes ^16^ or turn to private care, which can introduce corruption into the private sector as well.^65^ A possible explanation for these findings is the dual employment of healthcare workers in both public and private sectors, with unethical behaviors transferring between the two. Research on dual practice has shown that it often leads to conflicts of interest, resulting in preferential treatment for health service users who can afford to pay more.^66,67^ Doctors may neglect their public sector duties to benefit from higher earnings in the private sector^23^ and sometimes redirect health service users from public to private healthcare.^68^ Further investigation is needed to explore these dynamics.

 This cross-sectional study has several limitations. First, it only sampled participants from two states, which limits the ability to generalise the findings to all of Nigeria. Future research should include additional regions, such as the southern and other geographical areas, to capture a broader, more representative sample. Expanding the sample would enhance the validity and applicability of the findings by better reflecting the diversity of the population. The data in this study primarily focused on users’ general experiences of corruption when seeking healthcare in public health facilities. However, we only examined one form of corruption—bribery or “unapproved payments.” Future research should explore other types of corruption to provide a more comprehensive understanding of the factors driving different forms of corruption. In addition, researchers should consider further investigation to understand which healthcare services are more prone to corrupt practices. This would help develop more targeted interventions to address each type of corruption.

 Another limitation is using participants’ perceived sufficiency of household income as an independent variable rather than using an asset score. While an asset score reflects accumulated wealth over time, it may not capture recent financial changes. In contrast, the perceived sufficiency of income is a subjective measure that accounts for an individual’s current economic situation, including in-kind support and shared resources within social or kin networks, which are often difficult to capture through income categories alone. This measure offers insight into how individuals feel about their financial adequacy or inadequacy, reflecting their immediate economic experience.

## Conclusions

 Our study found that corruption impacts individuals differently based on various socio-demographic factors, including age, gender, geographic location, education level, perceived household income, and the type of healthcare facility used. These characteristics significantly influence how health service users experience corruption when seeking health services. Corruption in healthcare settings leads to unequal access to services, poor quality care, and higher out-of-pocket costs for health service users.

 To ensure that all social groups have access to quality, corruption-free healthcare, addressing corruption at the service delivery level, particularly “unapproved payments,” is crucial. Any interventions must take into account the diverse needs and challenges faced by different health service users. Potential solutions include targeted ethics training for healthcare providers, public awareness campaigns, and the integration of digital payment systems to reduce cash-based transactions, all of which could help reduce corruption and its disparities.

## Acknowledgements

 We thank the research assistants for their participation in data collection. We are grateful to all the respondents for giving us their time and participating in the study. We also acknowledge the acountability in action project team, Nigeria, for their contribution in the project that produced the data presented in this study.

## Ethical issues

 Ethical approval was obtained from the Health Research and Ethics Committee of the University of Nigeria Teaching Hospital Eastern State with reference number NHREC/05/01/2008B-FWA00002458-1RB00002323, and the London School of Hygiene and Tropical Medicine, UK (LSHTM ref:14 540-1). Written consent was obtained from each respondent following a detailed oral description (including the benefits and risks of participation and the study objectives) and a printed information sheet in the relevant language. The understanding was established before proceeding with the interview. Participation was voluntary, and respondents were informed that they could decline or withdraw at any stage of the interview. Confidentiality was assured, and personally identifiable data (such as the names of respondents) were assigned unique codes. In light of the sensitive nature of our quantitative data, researchers implemented stringent storage and safeguarding protocols to ensure its security.

## Conflicts of interest

 Authors declare that they have no conflicts of interest.
